# A multi-agent vision-language debate framework for zero-shot crop disease diagnosis

**DOI:** 10.3389/fpls.2026.1890016

**Published:** 2026-08-11

**Authors:** Mustafa Al Juboori, Zeeshan Abbas, Zayed Al Aghbari, Farman Ullah, Mobeen Ur Rehman

**Affiliations:** 1 College of Information Technology, United Arab Emirates University, Al-Ain, Abu Dhabi, United Arab Emirates; 2 Department of Biomedical Engineering, College of IT Convergence, Gachon University, Seongnam, Republic of Korea

**Keywords:** agricultural AI, collaborative reasoning, consensus learning, crop disease diagnosis, multi-agent AI, multi-agent deliberation, plant disease detection, zero-shot learning

## Abstract

Accurate crop disease diagnosis is critical for agricultural productivity and food security, yet existing deep learning systems often struggle to generalize across visually similar diseases and varying environmental conditions. Recent Vision-Language Models (VLMs) have demonstrated promising zero-shot reasoning capabilities; however, most agricultural diagnostic systems still rely on isolated single-model predictions without collaborative reasoning or consensus mechanisms. In this work, we propose VIDA+PANDA, a multi-agent Vision-Language framework for zero-shot crop disease diagnosis. The framework consists of two stages: VIDA, where multiple VLM agents independently analyze crop leaf images to establish baseline performance, and the Peer-Anchored Named Deliberation Architecture (PANDA), which introduces a structured multi-round debate among a selected group of high-performing and architecturally diverse agents. During deliberation, agents exchange reasoning, critique peer predictions, and revise decisions through evidence-grounded discussion, while an anti-sycophancy mechanism discourages unsupported consensus shifts. Final predictions are generated through performance-weighted consensus voting. Experiments are conducted on the CDDM benchmark using seven heterogeneous VLMs from four independent providers, including two open-source models, under a fully zero-shot setting. A non-participant GPT-5 model serves as an independent judge to assess the final diagnostic predictions. Beyond conventional accuracy, the framework introduces three semantic measures: Semantic Label Similarity (SLS), which measures how semantically close a predicted crop-disease pair is to the ground truth and captures partial correctness overlooked by exact-match evaluation; Reasoning Specificity (RS), which measures how concretely an agent’s explanation references visual evidence such as lesion color, shape, texture, or margins; and Inter-Agent Reasoning Convergence (IRC), which measures the extent to which agents rely on similar visual evidence, capturing epistemic alignment independently of label correctness. Experimental results show that collaborative multiagent deliberation improves individual diagnostic performance and semantic alignment, with the largest gains observed among weaker participating agents. The findings also reveal important relationships between predictive accuracy, persuasive influence, and consensus formation in VLM-based agricultural diagnosis.

## 1 Introduction

Agriculture remains central to global food security, yet the sector is increasingly challenged by climate change, biodiversity loss, labor shortages, and rapidly evolving plant pathogens. The global population is projected to exceed nine billion by mid-century, substantially increasing food demand and intensifying pressure on agricultural systems to improve productivity and sustainability (Nations, 2024). Simultaneously, rural-to-urban migration has contributed to labor shortages in agricultural regions (Hill et al., 2021), while environmental stressors such as global warming, soil degradation, and extreme climate events continue to destabilize crop production (Yang et al., 2024; Hasegawa et al., 2021). Rising temperatures and changing precipitation patterns are also accelerating the spread and emergence of crop diseases, increasing disease pressure across diverse agro-ecosystems (Pfenning-Butterworth et al., 2024; Kumar and Mukhopadhyay, 2025). These challenges collectively highlight the urgent need for rapid, scalable, and accurate crop disease diagnosis systems capable of supporting timely agricultural intervention.

Crop diseases and pests remain among the most significant threats to agricultural productivity, accounting for nearly 30% of global crop losses annually (Junaid and Gokce, 2024). Early and accurate disease diagnosis is therefore essential for minimizing yield losses, improving food supply chains, and supporting sustainable farming practices. These objectives align with the broader role of AI in addressing sustainability challenges and contributing to long-term development goals (Gao et al., 2026). Traditional disease identification methods typically rely on visual inspection by agricultural experts or laboratory-based analyses of infected plant tissues (Ahmad et al., 2023). Although these approaches can achieve high diagnostic accuracy, they are often labor-intensive, time-consuming, expensive, and impractical for large-scale deployment, particularly in resource-constrained regions where expert availability is limited. The increasing complexity and variability of disease symptoms under diverse environmental conditions further complicate reliable manual diagnosis.

Recent advances in precision agriculture have enabled the integration of sensing technologies, robotics, remote monitoring, and intelligent computing into modern farming workflows (Mgendi, 2024). Remote sensing platforms such as drones and satellites can capture large-scale field information in real time, while multispectral and hyperspectral imaging technologies provide detailed insights into plant physiological conditions (Dhaliwal et al., 2024; Rayhana et al., 2023; Wu et al., 2025). Within this context, artificial intelligence (AI), particularly computer vision-based deep learning, has emerged as a promising tool for automated crop disease diagnosis. AI-driven image analysis systems can identify subtle disease symptoms, including discoloration, lesion morphology, and texture abnormalities, at a speed and scale beyond human capability (Khan et al., 2024). Such systems also enable targeted pesticide application, thereby reducing excessive chemical usage and minimizing environmental and health risks.

Over the past decade, deep learning techniques have demonstrated remarkable progress in agricultural disease detection (Fu et al., 2023). Convolutional Neural Networks (CNNs) have been widely adopted for image-based disease classification tasks due to their ability to learn discriminative visual patterns directly from large image datasets (Beikmohammadi et al., 2022; Liu et al., 2024c). Numerous studies have reported strong performance using CNN-based architectures for crop disease recognition under controlled conditions. More recently, object detection frameworks such as YOLO have gained popularity because of their real-time inference capability and efficient deployment on edge and mobile devices, making them suitable for practical agricultural monitoring scenarios (Wang and Liao, 2024). Similar computer vision architectures have also demonstrated strong performance in automated inspection and anomaly detection applications, highlighting their ability to identify subtle visual patterns under real-world operating conditions (Xu et al., 2024). Vision Transformers (ViTs) have emerged as a powerful alternative to conventional CNNs by leveraging self-attention mechanisms to capture long-range spatial dependencies and global contextual relationships among image features (Dosovitskiy et al., 2020). Recent vision systems have further demonstrated the effectiveness of attention-based feature modeling for improving visual representation quality and supporting reliable inspection and recognition in complex imaging environments (Zhu et al., 2025b; Xu et al., 2025). Compared with traditional CNN architectures, ViTs demonstrate stronger capability in modeling complex lesion structures, irregular disease patterns, and distributed visual cues across plant leaves (Marwaha et al., 2023).

Despite these advances, existing deep learning approaches continue to face substantial limitations in real-world agricultural environments. Most supervised disease detection models rely heavily on large annotated datasets and often exhibit poor generalization when deployed outside their training distribution (Zhou et al., 2022). Environmental variability, particularly changes in illumination conditions, remains a major challenge for robust visual recognition systems (Xue et al., 2025). Variations in background complexity, geographic conditions, climate, crop variety, and disease morphology can further introduce severe domain shifts that significantly degrade model performance (Upadhyay et al., 2025; Duhan et al., 2026). Similar challenges have been observed in other vision-based systems operating in complex environments, where occlusion and visual ambiguity can substantially affect recognition and decision-making performance (Li et al., 2024). Moreover, visually similar diseases frequently produce overlapping symptoms, making fine-grained classification highly challenging even for advanced architectures (Ewieda et al., 2026). While CNNs and ViTs improve feature representation, they remain fundamentally dependent on static training distributions and single-model inference paradigms, limiting their adaptability and robustness under open-world agricultural conditions.

Recently, large Vision-Language Models (VLMs) have emerged as a promising paradigm for visual reasoning tasks by combining visual understanding with the semantic reasoning capabilities of large language models (Li et al., 2025). Unlike conventional supervised classifiers, VLMs demonstrate strong zero-shot and open-vocabulary recognition capabilities, enabling them to reason about unseen categories using natural language prompts and contextual knowledge (Allgeuer et al., 2025; Dong et al., 2025). These properties make VLMs particularly attractive for agricultural disease diagnosis, where collecting large-scale labeled datasets for every crop-disease combination is often impractical (Zhu et al., 2025a). However, individual VLMs still suffer from hallucination, inconsistent reasoning, overconfidence, and unstable decision-making, particularly when confronted with visually ambiguous disease symptoms (Xia et al., 2025). Furthermore, existing agricultural AI systems predominantly rely on isolated single-model predictions and rarely exploit collaborative reasoning among multiple AI agents.

Beyond general purpose VLMs, recent research has begun developing domain specific vision-language foundation models and benchmarks for agriculture. SCOLD introduces a vision-language foundation model trained on more than 186,000 image-text pairs for leaf disease identification and demonstrates strong few-shot and zero-shot performance through domain-adaptive contrastive learning (Quoc et al., 2025). AgriCLIP extends CLIP style pretraining to agriculture and livestock by constructing the large scale ALive dataset containing approximately 600,000 image-text pairs and learning domain-specialized visual-semantic representations (Nawaz et al., 2025). Concurrently, benchmark efforts such as CDDM, AGMMU, and LeafNet have highlighted the growing need for multimodal datasets that evaluate agricultural reasoning beyond conventional image classification, incorporating question answering, disease diagnosis, and knowledge intensive agricultural understanding (Liu et al., 2024b; Gauba et al., 2026; Quoc et al., 2026). Collectively, these studies demonstrate substantial progress in agricultural foundation models and multimodal reasoning. However, they primarily focus on improving the capabilities of individual models and rarely investigate whether multiple VLMs can collaboratively reason, challenge one another’s conclusions, and arrive at more reliable diagnoses through structured deliberation.

Inspired by recent advances in multi-agent AI systems and debate-based reasoning frameworks, this work introduces VIDA+PANDA, a multi-agent vision-language deliberation framework for zero-shot crop disease diagnosis. The proposed framework combines Varied Independent Diagnostic Analysis (VIDA) with the Peer-Anchored Named Deliberation Architecture (PANDA) to enable structured peer-aware reasoning among multiple VLM agents. In the VIDA stage, heterogeneous VLM agents independently analyze crop images to establish baseline diagnostic performance and characterize prediction diversity across agents. Subsequently, the PANDA stage conducts a multi-round named debate process in which agents exchange predictions, critique peer reasoning, and refine their diagnoses through evidence grounded deliberation. A performance-weighted consensus mechanism then aggregates the final predictions while reducing blind agreement and unsupported consensus shifts. Beyond conventional accuracy metrics, the framework also examines how collaborative deliberation influences semantic reasoning quality, interagent reasoning convergence, and epistemic alignment. Through this analysis, the study provides new insights into the benefits and limitations of multi-agent reasoning for agricultural diagnosis under zero-shot conditions.

## 2 Related work

### 2.1 Crop disease identification using machine learning

Early plant disease diagnosis methods primarily relied on manual field inspection and expert-driven visual assessment. Although these approaches can provide reliable diagnosis under controlled settings, they are often labor-intensive, time-consuming, and difficult to scale for large agricultural environments (Kotwal et al., 2023). In addition, manual diagnosis is highly dependent on domain expertise and may be affected by environmental conditions, symptom variability, and subjective interpretation. These limitations motivated the development of automated plant disease detection systems based on machine learning and deep learning techniques.

Traditional machine learning approaches such as Support Vector Machines (SVMs), Random Forests (RF), Decision Trees (DTs), Naive Bayes (NB), and K-Nearest Neighbors (KNNs) have been widely explored for crop disease classification tasks. Panigrahi et al. evaluated multiple classical machine learning algorithms for maize disease detection and reported that RF achieved the highest classification accuracy among the evaluated models (Panigrahi et al., 2020). Similarly, Kaur et al. combined Fractional-order Zernike Moments (FZM) with SVM classifiers for grape leaf disease recognition (Kaur et al., 2019), while Yousuf and Khan (2021) utilized ensemble learning approaches based on RF and KNN for plant disease detection (Yousuf and Khan, 2021). Although these methods demonstrated encouraging performance in controlled experimental settings, conventional machine learning pipelines typically require handcrafted feature extraction and often struggle with high-dimensional image representations, multiclass disease recognition, and generalization under diverse environmental conditions.

With the rapid advancement of deep learning, Convolutional Neural Networks (CNNs) have become one of the dominant approaches for automated plant disease diagnosis due to their ability to learn hierarchical visual representations directly from raw image data. Several studies have reported strong performance using CNN-based architectures for crop disease classification tasks (Khan et al., 2023). For example, Shelar et al. proposed a CNN-based plant leaf disease classification framework covering 15 disease categories and achieved promising diagnostic accuracy (Shelar et al., 2022). Compared with traditional machine learning methods, CNNs eliminate the need for manual feature engineering and generally exhibit stronger robustness against variations in texture, color, and lesion morphology.

More recently, advanced deep learning architectures such as Variational Autoencoders (VAEs) and Vision Transformers (ViTs) have further expanded the capabilities of agricultural disease detection systems. VAEs have been explored for feature extraction, dimensionality reduction, and latent representation learning in complex agricultural datasets. Majikumna et al. integrated focal-loss VAEs with Grey Wolf Optimization-based CNNs for olive leaf disease classification (Majikumna et al., 2024), while Nagachandrika et al. proposed a multi-scale adaptive framework combining VAE-based feature learning with deep CNN architectures for robust plant disease recognition under varying visual conditions (Nagachandrika et al., 2024).

In parallel, Vision Transformers have emerged as a powerful alternative to conventional CNN architectures for image understanding tasks. Unlike CNNs, which primarily focus on local receptive fields, ViTs employ self-attention mechanisms to model long-range spatial dependencies and global contextual relationships within images. This capability is particularly beneficial for crop disease diagnosis, where disease symptoms may appear as subtle, irregular, or spatially distributed lesion patterns. De Silva and Brown demonstrated the effectiveness of ViTs for multispectral plant disease detection (De Silva and Brown, 2023), while Barman et al. reported strong performance of transformer-based architectures in complex agricultural environments with noisy and heterogeneous datasets (Barman et al., 2024).

Several recent studies have also explored hybrid architectures that combine CNNs, transformers, and latent feature modeling techniques to improve classification robustness. Ahmed et al. introduced a lightweight transformer assisted CNN framework based on a modified BEIT architecture for rice disease recognition (Ahmed et al., 2024). Similarly, Faisal et al. proposed a hybrid feature fusion strategy integrating Swin Transformers, VAEs, and MobileNetV3 for coffee leaf disease detection (Faisal et al., 2023). Few-shot learning approaches have also gained attention in scenarios with limited labeled data. Boulila et al. proposed a few-shot learning framework combining Swin Transformers with performer-attention mechanisms for efficient disease recognition using limited training samples (Boulila, 2024). In addition, Liu et al. introduced PMLPNet, a patch-based neural architecture designed for fine-grained multiclass plant disease classification through spatial and channel-aware contextual feature modeling (Liu et al., 2024a).

Despite these advances, existing crop disease detection approaches still face several important limitations. Many deep learning models remain highly dependent on large annotated datasets and supervised training pipelines, making them difficult to generalize across unseen crops, environmental conditions, and disease categories. Furthermore, most existing systems rely on single-model inference strategies and do not explicitly incorporate collaborative reasoning, semantic deliberation, or multi-agent consensus mechanisms during diagnosis. These limitations become particularly significant in zero-shot and open-world agricultural scenarios, where disease symptoms may exhibit substantial visual ambiguity and limited labeled training data may be available.

### 2.2 Large Language Models and Vision-Language Models in agriculture

Recent advances in Large Language Models (LLMs) and Vision-Language Models (VLMs) have introduced new opportunities for intelligent agricultural analysis and decision support. Unlike conventional deep learning models that primarily operate on fixed classification objectives, LLMs provide strong semantic reasoning, contextual understanding, and natural language generation capabilities, enabling more interactive and explainable AI systems in agricultural applications (Tian et al., 2025; Kang et al., 2025). These models can assist in generating disease management recommendations, interpreting diagnostic outcomes, and supporting agricultural decision-making processes through natural language interaction.

Several recent studies have explored the integration of LLMs into agricultural disease diagnosis pipelines. Karimanzira proposed a tomato disease detection framework that combined Vision Transformers with cascaded attention mechanisms and incorporated LLMs to generate interpretable diagnostic explanations and disease management recommendations (Karimanzira, 2025). Similarly, Jiang et al. addressed the limitations of general-purpose LLMs in agricultural applications by constructing a domain-specific agricultural question-answering dataset and employing knowledge-guided fine-tuning together with retrieval-enhanced self-reflection strategies to improve response quality and domain consistency (Jiang et al., 2025). Wang and Zhao proposed an agricultural knowledge graph framework based on natural language processing and deep learning for structured agricultural knowledge representation and downstream intelligent search applications (Wang and Zhao, 2024). In addition, Chen and Huang investigated the integration of reinforcement learning and LLMs for crop management decision support systems, demonstrating the potential of combining adaptive optimization with semantic agricultural reasoning (Chen and Huang, 2025).

Alongside LLMs, VLMs have recently emerged as a promising paradigm for multimodal agricultural understanding by jointly modeling visual and textual information (Zhu et al., 2025a). VLMs extend the capabilities of traditional image classification systems by enabling open-vocabulary recognition, contextual visual reasoning, and zero-shot inference through natural language prompting (Allgeuer et al., 2025). Recent studies have further demonstrated the potential of VLMs for zero-shot agricultural perception and reasoning tasks, including weed detection in UAV-based precision agriculture settings (Nasir et al., 2026). Recent multimodal agricultural systems have further demonstrated the potential of large vision-language architectures for fine-grained agricultural understanding tasks, including pest recognition and segmentation under complex field conditions (Yang et al., 2026). These properties are particularly attractive for agricultural disease diagnosis, where collecting large-scale labeled datasets for every crop and disease category is often impractical. Furthermore, VLMs provide greater flexibility for handling unseen diseases, ambiguous visual symptoms, and semantically complex diagnostic tasks compared with conventional supervised classifiers.

Despite their potential, current agricultural applications of LLMs and VLMs remain relatively limited and primarily focus on single-model inference or text-based advisory systems. Existing approaches rarely investigate collaborative reasoning, peer-aware deliberation, or consensus-based decision-making among multiple VLM agents. In addition, individual VLMs may still suffer from hallucination, inconsistent reasoning, and unstable predictions when confronted with visually similar disease categories or ambiguous lesion patterns. These limitations highlight the need for more robust multi-agent reasoning frameworks capable of leveraging diverse model perspectives while improving interpretability and diagnostic reliability in zero-shot agricultural environments.

Recent agricultural foundation-model research has also focused on large-scale multimodal datasets and domain-specialized pretraining. CDDM introduced one of the earliest crop-disease multimodal benchmarks, comprising approximately 137,000 disease images and one million question–answer pairs designed for agricultural conversational AI (Liu et al., 2024b). More recently, AGMMU proposed a knowledge-intensive benchmark derived from expert–grower interactions, while LeafNet introduced a large-scale multimodal plant pathology dataset containing 186,000 images and rich expert-annotated metadata for evaluating VLMs on disease understanding tasks (Gauba et al., 2026; Quoc et al., 2026). In parallel, domain-specific foundation models such as AgriCLIP and SCOLD have demonstrated that agricultural pretraining can substantially improve zero-shot and few-shot performance compared with general-purpose vision-language models (Nawaz et al., 2025; Quoc et al., 2025). Large-scale biological foundation models such as BioCLIP2 further demonstrate the value of domain-specific contrastive pretraining for fine-grained biological visual understanding (Gu et al., 2026). These studies collectively highlight the increasing importance of agricultural and biological foundation models and multimodal benchmarks. However, they primarily evaluate individual models and do not investigate collaborative deliberation, peer influence, or consensus formation among multiple VLM agents.

Motivated by these limitations, the present work investigates collaborative multi-agent deliberation among heterogeneous VLMs for zero-shot crop disease diagnosis. Unlike existing agricultural AI systems that primarily rely on isolated predictions from individual models, the proposed VIDA+PANDA framework enables structured peer-aware debate, evidence-grounded reasoning exchange, and performance-weighted consensus formation among multiple VLM agents. In addition to evaluating diagnostic accuracy, the framework examines how collaborative deliberation influences reasoning quality, semantic alignment, and inter-agent agreement. By doing so, this study provides a systematic investigation of multi-agent reasoning as a mechanism for improving the robustness and interpretability of zero-shot agricultural diagnosis.

## 3 Benchmark datasets

We evaluate the proposed framework using the Crop Disease Diagnosis Multi-Modal (CDDM) benchmark introduced by Liu et al. (2024b). The benchmark covers 54 disease categories across 16 crop species and presents a challenging fine-grained recognition task due to the substantial visual similarity between diseases affecting the same crop (*e.g.*, Apple Scab, Apple Cedar Apple Rust, and Apple Black Rot).

The primary objective of this work is to study multi-agent deliberation dynamics, including peer influence, semantic convergence, and consensus formation. As each image is processed through the VIDA stage followed by multi-round PANDA deliberation across multiple agents, the evaluation pipeline requires repeated large-scale VLM inference and is computationally intensive. To keep the evaluation controlled, reproducible, and tractable, we construct a stratified subset by randomly sampling up to 25 images per class using a fixed seed of 42. The resulting pool is adjusted to an approximate 80% diseased and 20% healthy distribution to better reflect a realistic healthy-to-diseased class balance. The panel-selection ablation reported in Section 5.3 was conducted on an earlier exploratory subset comprising 490 images, whereas the primary evaluation dataset used in this study contains 1,219 images. The larger sample was selected to improve statistical stability and to assess whether the observations from the exploratory study generalize to a broader evaluation set.

The resulting evaluation set contains 1,219 images spanning 53 of the 54 available classes (the Tomato Yellow Leaf Curl category was not present in the accessible partition and is therefore excluded). All VIDA and PANDA configurations are evaluated on this identical set to ensure fair and directly comparable analysis. The dataset name is withheld from all model prompts (Section 4.2) to eliminate the possibility that a foundation model recognizes the benchmark by name rather than reasoning directly from the image.

## 4 Methodology

### 4.1 Overview

**Figure 1:**
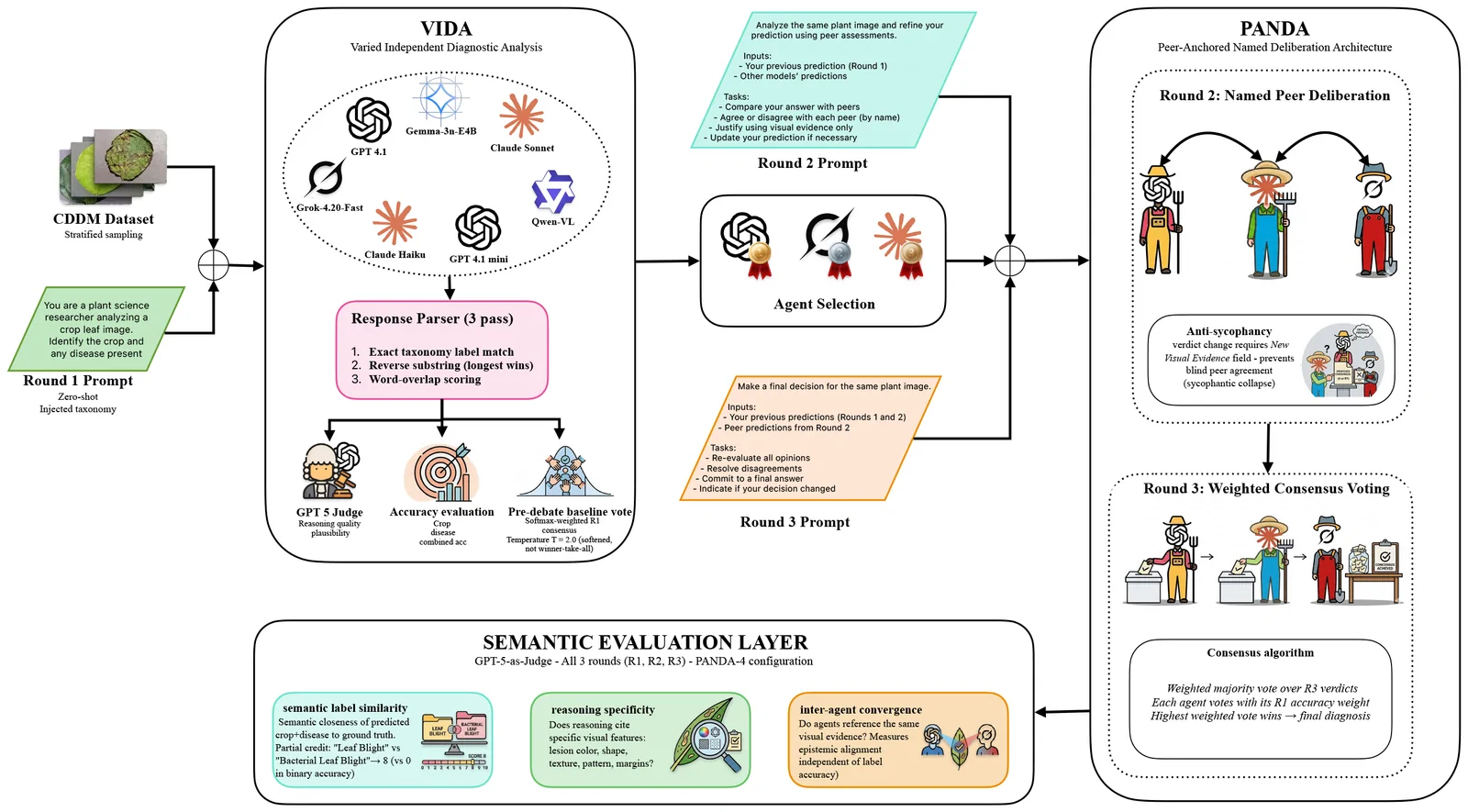
Overview of the proposed VIDA+PANDA framework for zero-shot multi-agent crop disease diagnosis. Leaf images from the CDDM benchmark are combined with a structured zero-shot prompt containing the complete taxonomy to constrain all agents to valid crop and disease labels. The dataset name is withheld from the prompt to prevent benchmark-identity leakage. In the VIDA stage, seven VLMs, including two open-source models, independently analyze each image without peer interaction. Responses are processed by a three-stage parser and scored by an independent, non-participant GPT-5 judge for reasoning quality and plausibility. A softmax-weighted baseline consensus is computed from the Round 1 predictions. Based on VIDA performance and model diversity, a subset of agents is selected to enter the PANDA stage for a two-round named peer debate. Agents may revise a decision only when supported by new visual evidence, reducing blind agreement and sycophantic behavior. Final predictions are aggregated through a softmax-weighted consensus, while SLS, RS, and IRC are used to evaluate semantic correctness, reasoning quality, and inter-agent alignment.

We propose VIDA+PANDA, a two-stage multi-agent framework for zero-shot crop disease diagnosis using VLMs. The first stage, termed VIDA, evaluates all participating agents independently on each input image to establish baseline diagnostic performance and characterize prediction diversity across agents. The second stage, referred to as PANDA, introduces a structured multi-agent debate process in which a selected subset of high-performing and architecturally diverse agents exchange reasoning, critique peer predictions, and iteratively refine their decisions through named deliberation. The final diagnosis is generated through a performance-weighted consensus mechanism based on the debate outcomes. An overview of the complete VIDA+PANDA pipeline is presented in **Figure 1**.

The proposed framework operates entirely in a zero-shot setting, without task-specific fine-tuning, annotated training samples, or few-shot demonstrations. Each agent receives only the input crop image together with a structured prompt containing the complete disease taxonomy. This setup enables the framework to evaluate the zero-shot reasoning and generalization capabilities of VLMs for fine-grained crop disease diagnosis under realistic open-world conditions.

### 4.2 Agent configuration

Seven VLMs from four independent providers are used throughout the experiments (**Table 1**). Incorporating models from different organizations is an intentional design choice. Because these models are trained on different data, architectures, and alignment strategies, they are less likely to share the same failure modes, reducing the risk of correlated errors during consensus formation. To improve reproducibility and explore lower-cost deployment settings, the lineup includes two open-source agents served through Together.ai, Gemma-3n-E4B and Qwen3.5-9B (denoted Qwen-VL), alongside proprietary models. This allows the framework to be evaluated using a mixture of open-source and commercial VLMs.

**Table 1:** Agent lineup used in the VIDA block (*N* = 1,219 images).

Agent	Provider	Model string	Crop acc.	Combined acc.
GPT-4.1	OpenAI	gpt-4.1	0.837	0.366
Grok-4.20-Fast	xAI	grok-4.20-non-reasoning	0.728	0.326
GPT-4.1-mini	OpenAI	gpt-4.1-mini	0.684	0.303
Claude Sonnet 4.5	Anthropic	claude-sonnet-4-5	0.526	0.186
Qwen-VL (open)	Together.ai	Qwen/Qwen3.5-9B	0.652	0.184
Claude Haiku 4.5	Anthropic	claude-haiku-4-5	0.418	0.121
Gemma-3n-E4B (open)	Together.ai	google/gemma-3n-E4B-it	0.591	0.098

Two open-source models (Gemma3n-E4B and Qwen-VL) are included alongside proprietary agents to improve accessibility and support more reproducible evaluations. Combined Accuracy requires both the crop and disease labels to be correct. Confidence intervals and calibration statistics are reported in **Table 2**.

To minimize taxonomy hallucination, all agents are constrained by a structured system instruction that lists the valid crop and disease labels and requires the model to select from that taxonomy. Unlike our earlier configuration, the prompt does not include the name of the source dataset. Instead, each model is instructed to operate as a generic plant pathologist:

“You are an expert plant pathologist classifying crop leaf images. Valid crops (choose EXACTLY one): [full list]. Valid diseases (choose EXACTLY one): [full list]. NEVER output a crop or disease not in these lists. If unsure, pick the closest match. Do NOT ask for clarification always classify.”

Withholding the benchmark name removes any cue that might allow a pretrained model to rely on dataset specific associations rather than reasoning directly from the image. This reduces a potential source of benchmark leakage and provides a more reliable assessment of zero-shot diagnostic capability. All results reported in this work use the name-withheld configuration.

The system prompt is delivered through each provider’s native interface, and all agents are allocated a maximum output budget of approximately 600–700 tokens. For the open-source Qwen3.5-9B agent, the provider’s default chained-reasoning (“thinking”) mode is disabled. This ensures that the model produces a direct structured response rather than consuming part of its output budget on hidden reasoning traces.

### 4.3 Varied Independent Diagnostic Analysis

In the VIDA block, all seven agents independently analyze each image with no access to peer outputs.

Each agent receives a structured prompt requesting:

Crop Category: <one of the valid crops>
Disease Category: <one of the valid diseases>
Diseased: <Yes or No>
Reasoning: < 2–3 sentences citing specific visual features>
Confidence: < 0.0 to 1.0>

Responses are aligned to the taxonomy with a three-stage parser: (i) exact string match after case normalization, (ii) reverse-substring matching for truncated outputs (*e.g.*, “Gray leaf spot” → “Cercospora leaf spot Gray leaf spot”), preferring the longest matching label to preserve specificity, and (iii) word-overlap scoring when the first two stages fail, with ties broken toward more descriptive labels.

Following VIDA, all responses are scored by an independent judge model on two dimensions: Reasoning Quality (RQ), which measures whether the response references meaningful visual evidence such as lesion color, shape, or texture, and Plausibility, which measures whether the diagnosis is scientifically reasonable even when incorrect. Both metrics are scored on a 1–10 scale. The judge is GPT-5, a model that does not participate in any debate configuration. This separation prevents any agent from evaluating its own outputs and avoids evaluator-participant overlap. GPT-5 is queried with an explicit completion-token budget and minimal reasoning effort. Automatic budget expansion is applied when necessary to ensure that every response receives a valid score.

Agents entering PANDA are selected based on their VIDA Combined Accuracy. To avoid redundancy among closely related model families and to promote architectural diversity during deliberation, only the strongest-performing representative from each model lineage is retained. As a result, although GPT-4.1-mini achieved a higher VIDA score than some candidates, it was excluded because GPT-4.1 was already selected. The final PANDA panel therefore balances diagnostic performance with model diversity. Consensus influence is assigned using softmax-normalized weights derived from VIDA Combined Accuracy with temperature *T* = 2.0:

(1) $$w_{i}\;=\;\frac{\text{exp}\;\left({\text{CombAcc}}_{i}\;/\;T\right)}{\sum_{j=1}^{N}\text{exp }\left({\text{CombAcc}}_{j}\;/\;T\right)},\;T=2.0.$$

A temperature of *T* = 2.0 produces a softened weighting scheme that favors stronger agents while avoiding winner-take-all behavior as *T* → 0. We use *N* = 3 debate agents, the minimum configuration that enables multilateral named deliberation while keeping computational cost manageable.

### 4.4 PANDA block: Peer-Anchored Named Deliberation Architecture

Structured interaction among multiple agents has been shown to facilitate information exchange and collective decision-making in multi-agent learning systems (Liu et al., 2025). Motivated by this principle, the PANDA block runs Rounds 2 and 3 of the deliberation process, following the independent Round 1 diagnosis generated during VIDA. Unlike standard ensemble approaches that aggregate predictions anonymously, PANDA agents are explicitly aware of each other’s identities, Round 1 verdicts, and reasoning throughout the debate.

#### 4.4.1 Round 2 - named peer debate

In the second debate round, each agent receives its own Round 1 prediction together with the predictions and abbreviated reasoning summaries of all peer agents. Unlike conventional ensembles, PANDA enables direct peer-aware interaction, allowing agents to analyze, support, or challenge one another’s conclusions using visual evidence from the image.

The Round 2 prompt instructs agents to address peers by name, explain whether they agree or disagree with peer predictions, and revise their diagnosis only when new visual evidence can be identified beyond what was stated in Round 1. This encourages evidence-grounded reasoning rather than blind consensus formation.

To support analysis of debate dynamics, Round 2 responses extend the original prediction format with two additional fields:

- Influenced By: identifies the peer agent responsible for a prediction change, or None if the original verdict is maintained.
- New Visual Evidence: specifies the newly identified visual feature supporting the revised diagnosis, or NONE if no additional evidence is observed.

Requiring agents to explicitly provide new visual evidence before modifying their prediction serves as the primary anti-sycophancy mechanism of the framework. Responses that change labels without providing additional visual justification are flagged as unsupported agreement. This constraint helps reduce blind conformity toward stronger-performing agents, a failure mode referred to as sycophantic collapse.

#### 4.4.2 Round 3 - final verdict

During Round 3, agents receive their complete reasoning history from the previous rounds together with all peer responses from Round 2. Each agent then performs a final re-evaluation of the image and commits to an irreversible final diagnosis. The prompt again requires agents to explicitly reference peer opinions and justify any remaining disagreements or revised conclusions.

Round 3 follows the same response structure introduced in Round 2, allowing analysis of second-order influence patterns and late-stage reasoning shifts that emerge during collaborative deliberation.

#### 4.4.3 Consensus voting

To establish a reference point for evaluating the effect of deliberation, a pre-debate consensusprediction is first computed using the Round 1 outputs of the selected agents together with the samesoftmax weighting strategy defined in **Equation 1**. This ensures that any performance difference observed after deliberation is attributable to the debate process itself rather than changes in voting methodology.

After Round 3, the final framework prediction is generated through weighted consensus aggregationusing **Equation 2**:

(2) $$\hat{y}\;=\;\underset{c\;\in \;C}{\arg\max}\sum_{i=1}^{N}w_{i}\cdot 1\;[{\hat{y}}_{i}^{\left(3\right)}=c],$$

where C denotes the set of valid crop-disease labels and ${\hat{y}}_{i}^{\left(3\right)}$ represents the final Round 3 prediction of agent *i*.

### 4.5 Semantic evaluation

Exact-match accuracy penalizes “Leaf Blight” and “Bacterial Leaf Blight” equally despite their closeness. Because the framework studies collaborative reasoning, not only classification, we introduce three complementary semantic measures, scored by a judge over the PANDA debate transcripts of the exploratory pilot study.

Semantic Label Similarity (SLS) scores, on 0–10, how semantically close the predicted crop–disease pair is to the ground truth: exact matches score highest, while related predictions receive partial credit, capturing correctness that exact match ignores.

Reasoning Specificity (RS) scores, on 0-10, the visual detail of an explanation: responses citing precise lesion characteristics (color, texture, shape, margins, spatial distribution) score higher than vague reasoning, indicating whether debate yields more visually grounded explanations.

Inter-Agent Reasoning Convergence (IRC) measures the degree to which agents reference the samevisual evidence, focusing on epistemic alignment rather than label correctness. For round*R* the IRC is calculated using **Equation 3**,

(3) $$\text{IRC}(R)\;=\;J\;({\left\{r_{i}^{\left(R\right)}\right\}}_{i=1}^{N},\;\;A),$$

where $r_{i}^{\left(R\right)}$ is agent *i*’s reasoning at round *R*, A the rubric, and J the judge. All semantic scores use structured single-integer prompting and are reported as means with 95% confidence intervals.

The semantic analysis is conducted on the exploratory pilot study (Section 5.3), where the PANDA-4 configuration was evaluated using GPT-5 as the judge. By contrast, all primary accuracy, plausibility, and reasoning-quality results reported in the main study are evaluated using the independent, non-participant GPT-5 judge. The RS and IRC metrics focus on the specificity and convergence of agent reasoning rather than the correctness of the final prediction, making them less dependent on the judge’s own diagnostic preferences. Human expert validation of a representative subset of semantic scores remains an important direction for future work.

### 4.6 Implementation details

All experiments are conducted using commercial and open API endpoints without local model deployment, requiring only CPU resources and internet access. Images are transmitted as base64-encoded inputs for OpenAI and Anthropic models. For Together.ai models (Gemma-3n-E4B and Qwen-VL), images are first uploaded to a public URL because the serverless endpoints do not support inline base64 inputs. Checkpoints are written atomically at regular intervals to enable cross-session recovery without data loss or corruption.

The complete codebase, structured prompts, and raw API responses are available at https://github.com/mustafanfj/vida-panda-crop-disease.

For reproducibility, a fixed random seed of 42 is used for all stratified sampling procedures. Agent weights are computed deterministically from Round 1 Combined Accuracy. All providers are accessed through standard production endpoints. The GPT-5 judge is queried with an explicit completion-token budget and minimal reasoning effort, while the open-source Qwen agent is queried with chained-reasoning disabled. No preview or experimental model variants are used in any reported experiment.

## 5 Results

We first report independent VIDA performance of all seven agents at scale, then the dynamics and consensus behavior of the headline PANDA debate, followed by the agent-configuration ablation and the semantic analysis of reasoning convergence. Unless stated otherwise, all main results use the 1,219-image evaluation set with the dataset name withheld from prompts and a non-participant GPT-5 judge.

### 5.1 VIDA: independent diagnostic performance

**Figure 2:**
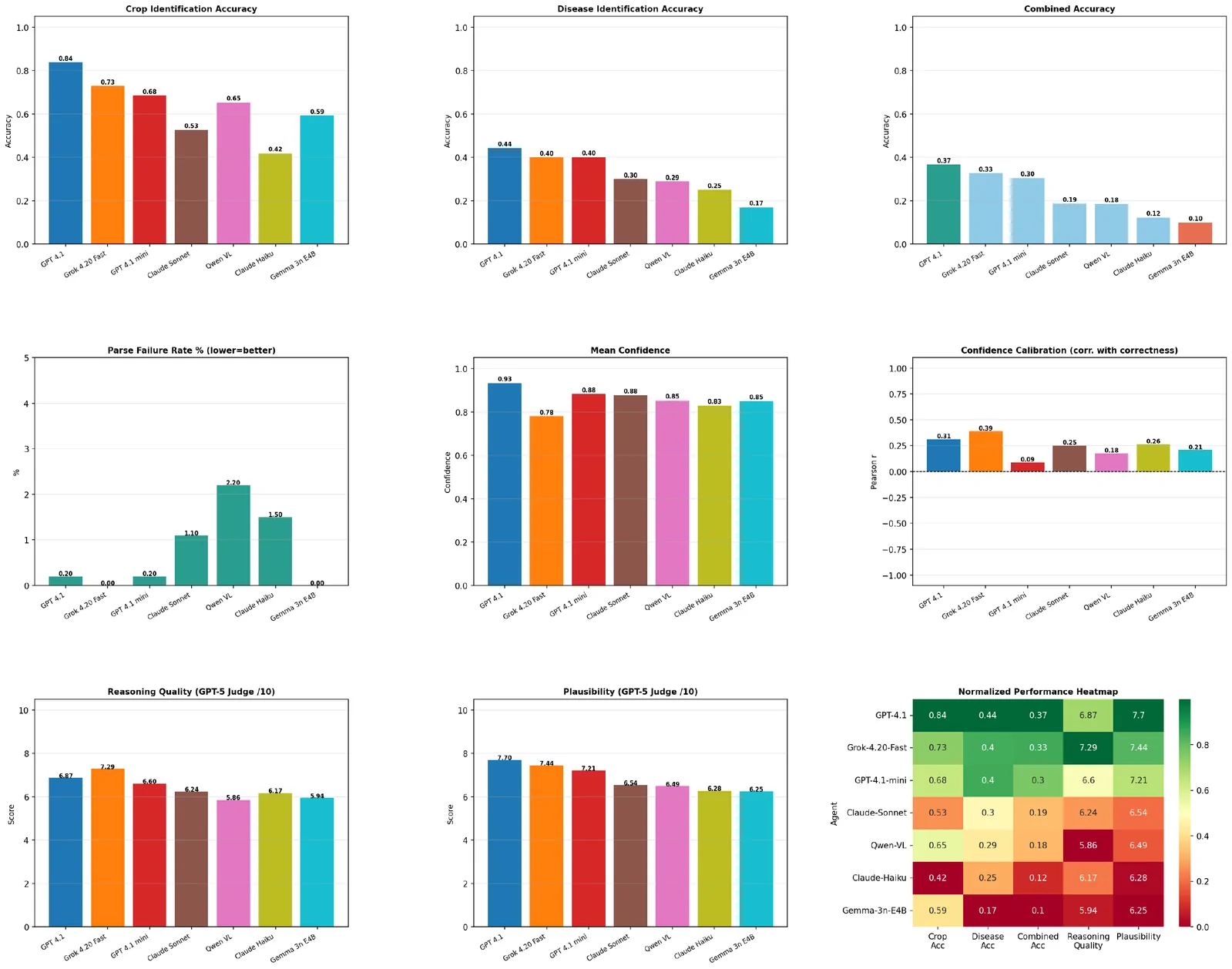
VIDA block dashboard across all seven agents on the 1,219-image CDDM evaluation set, sorted by combined accuracy. Top row: crop, disease, and combined accuracy. Middle row: parse-failure rate, mean confidence, and confidence calibration (Pearson *r* with correctness). Bottom row: GPT-5 judge scores for reasoning quality and plausibility, and a normalized performance heatmap. The dataset name is withheld from all prompts, and reasoning-quality and plausibility are scored by the non-participant GPT-5 judge.

**Figure 2** presents the nine-panel VIDA dashboard for all seven agents on the 1,219-image evaluation set, and **Table 2** reports the same per-agent accuracies with Wilson 95% confidence intervals, confidence correctness calibration, parse-failure rate, and the GPT-5 judge’s reasoning-quality (RQ) and plausibility (Plaus.) scores.

**Table 2:** VIDA Round-1 per-agent performance on the 1,219-image, 53-class evaluation set.

Agent	Combined	Crop	Disease	Wilson 95% CI	Calib. *r*	Parse%	RQ/Plaus.
GPT-4.1	0.366	0.837	0.441	[0.339,0.393]	0.314	0.16	6.87/7.70
Grok-4.20-Fast	0.326	0.728	0.399	[0.300,0.352]	0.389	0.00	7.29/7.44
GPT-4.1-mini	0.303	0.684	0.400	[0.278,0.329]	0.089	0.16	6.60/7.21
Claude-Sonnet	0.186	0.526	0.299	[0.165,0.209]	0.119	1.07	6.24/6.54
Qwen-VL	0.184	0.652	0.288	[0.163,0.206]	0.172	2.22	5.86/6.49
Claude-Haiku	0.121	0.418	0.250	[0.103,0.140]	0.185	1.48	6.17/6.28
Gemma-3n-E4B	0.098	0.591	0.169	[0.083,0.116]	0.208	0.00	5.94/6.25

Combined requires crop *and* disease simultaneously correct. Wilson 95% CIs are for Combined accuracy. Calib. *r* is the Pearson correlation between self-reported confidence and correctness. RQ and Plaus. are 1–10 scores from the non-participant GPT-5 judge.

GPT-4.1 leads on Combined accuracy (0.366) and crop accuracy (0.837), followed by Grok-4.20-Fast (0.326) and GPT-4.1-mini (0.303). Notably, the open-source Qwen-VL agent (0.184 Combined, 0.652 crop) lands mid-pack above Claude-Haiku and Gemma-3n-E4B confirming that a freely available model is competitive within the pool rather than uniformly weakest.

The judge scores are lower and more dispersed (RQ 5.86–7.29, Plaus. 6.25-7.70) than in our earlier pilot. This difference is consistent with the use of a stricter, independent judge model, which provides greater separation between fluent explanations and evidence-grounded reasoning. Calibration is weak across the board (Pearson *r* between 0.09 and 0.39), indicating that none of the agents’ confidence reliably tracks correctness an important caveat for any single-model deployment and part of the motivation for consensus.

#### 5.1.1 Per-crop difficulty

Performance varies enormously across crops (**Table 3**). A Kruskal–Wallis test confirms highly significant heterogeneity (*H* = 830.12, *p* ≈ 3.1×10^−167^). Visually distinctive crops such as Wheat (0.952), Raspberry (0.895), and Pumpkin (0.880) are diagnosed reliably, whereas crops with many confusable diseases Rice (0.127), Cherry (0.068), Peach (0.103), Apple (0.242) collapse even for the strongest agent.

**Table 3:** Per-crop combined accuracy.

Crop	*N*_img_	GPT-4.1	Best	Worst
Apple	264	0.242	0.242	0.053
Bell Pepper	43	0.558	0.791	0.000
Blueberry	17	0.471	0.529	0.000
Cherry	44	0.068	0.386	0.000
Corn	88	0.420	0.420	0.091
Grape	93	0.484	0.591	0.075
Orange	42	0.619	0.619	0.214
Peach	39	0.103	0.333	0.000
Potato	70	0.671	0.671	0.000
Pumpkin	25	0.880	0.960	0.080
Raspberry	19	0.895	0.895	0.053
Rice	150	0.127	0.307	0.000
Soybean	15	0.667	0.733	0.000
Strawberry	45	0.489	0.689	0.067
Tomato	244	0.320	0.320	0.029
Wheat	21	0.952	0.952	0.048

“Best”/”Worst” are the strongest/weakest single agent on that crop. Kruskal–Wallis across crops: *H* = 830.12, *p* ≈ 3.1 × 10^−167^.

#### 5.1.2 Per-disease difficulty

**Table 4** ranks diseases by Combined accuracy pooled across all seven agents. The hard classes share a clear signature: high *crop* accuracy but near-zero *disease* accuracy (e.g. Target Spot, crop 0.43/disease 0.00; Grey Spot, 0.91/0.01; Alternaria Blotch, 0.93/0.03). The agents correctly identify the plant but cannot resolve the specific lesion the bottleneck is fine-grained disease discrimination, not crop recognition. Crucially, scaling to 25 images per class reduced the number of zero-Combined-accuracy diseases from six (in the 490-image agent-selection ablation) to a single one (Target Spot), indicating that several of those zeros were small-sample artifacts rather than systematic blind spots. The healthy-versus-diseased gap is large (Combined 0.506 vs 0.156), implying that a binary disease-triage formulation is far more tractable than full fine-grained diagnosis.

**Table 4:** Hardest and easiest diseases by combined accuracy, pooled across all seven agents (*N*_img_ images per disease, ≥ 10).

Disease	*N*img	Crop	Disease	Combined
10 hardest				
Target Spot	25	0.429	0.000	0.000
Grey Spot	25	0.909	0.006	0.006
Leaf Smut	25	0.486	0.006	0.006
Bacterial Leaf Blight	25	0.469	0.040	0.029
Alternaria Blotch	25	0.926	0.034	0.034
Spider Mites	25	0.486	0.046	0.040
Leaf Rust	50	0.717	0.091	0.049
Leaf Spot	25	0.960	0.063	0.057
Scab	25	0.777	0.069	0.069
Leaf Mold	25	0.669	0.080	0.074
5 easiest				
Northern Leaf Blight	25	0.897	0.520	0.514
Healthy	244	0.634	0.775	0.506
Citrus Greening	25	0.629	0.343	0.343
Powdery Mildew	100	0.416	0.594	0.291
Early Blight	50	0.449	0.377	0.274

At 25 images/class only Target Spot records zero Combined accuracy, down from six in the 490-image pilot.

#### 5.1.3 Robustness to sample size

To verify that the reported statistics are not biased by sample size, we re-aggregated the identical pipeline on a stratified 490-image subsample drawn from the same run, holding every other factor fixed (**Table 5**). Per-agent Combined accuracy shifts by at most 0.014, comfortably within the Wilson CI widths of **Table 2**.

**Table 5:** Robustness to sample size: Combined accuracy on the full set versus a stratified 490-image subsample that holds all other factors fixed.

Agent	Full (1,219)	Subsample (490)	Δ
GPT-4.1	0.366	0.352	-0.014
Grok-4.20-Fast	0.326	0.319	-0.007
GPT-4.1-mini	0.303	0.288	-0.014
Claude-Sonnet	0.186	0.188	+0.002
Qwen-VL	0.184	0.188	+0.004
Claude-Haiku	0.121	0.125	+0.004
Gemma-3n-E4B	0.098	0.094	-0.004

All deltas fall within the Wilson CI widths of **Table 2**.

Because only *N* changes between the two columns, this is a clean test of the small-sample concern and shows the conclusions are stable.

### 5.2 PANDA: debate dynamics and consensus

**Figure 3:**
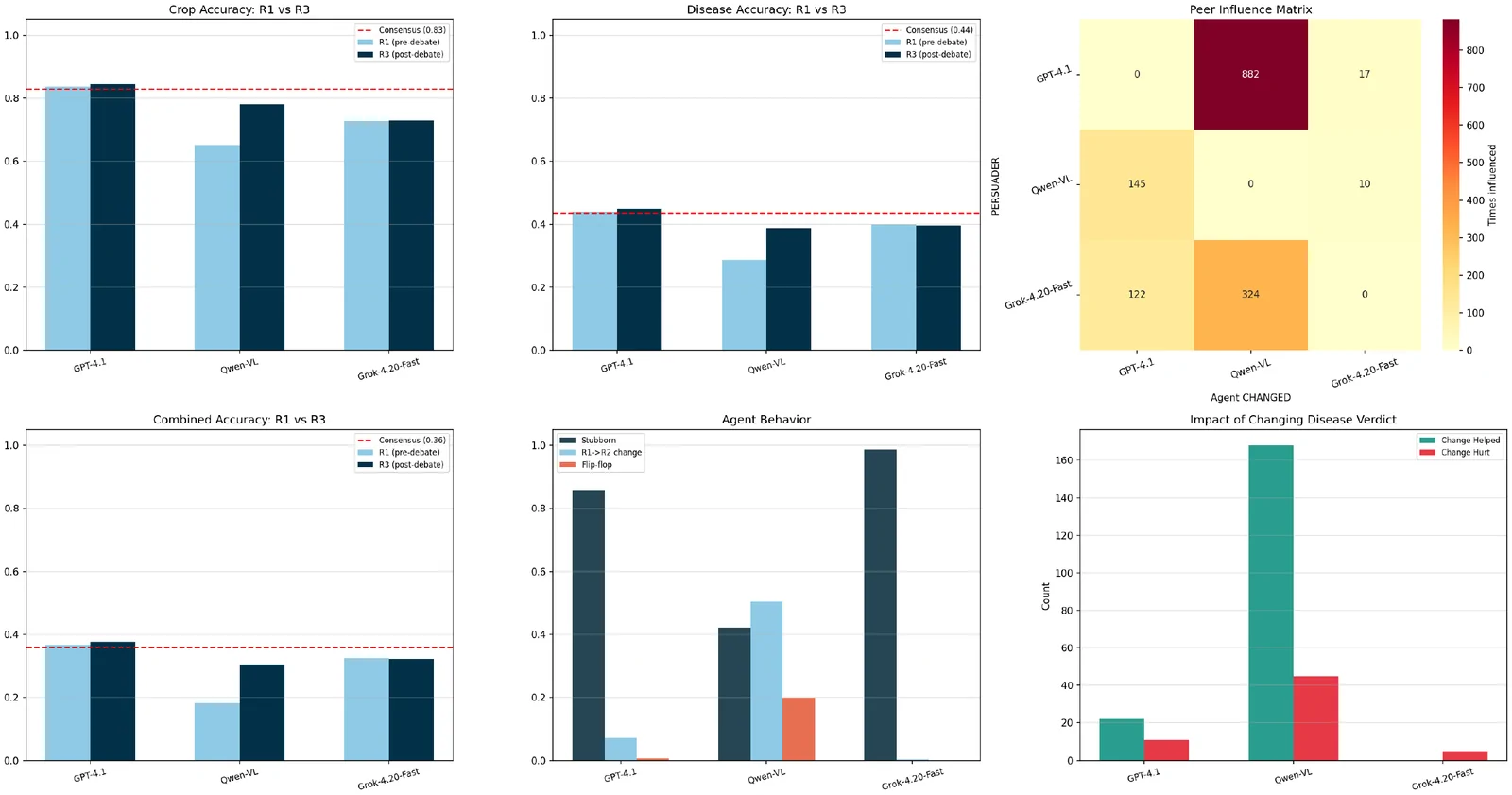
PANDA debate analysis for the headline panel {GPT-4.1, Qwen-VL, Grok-4.20-Fast} on the 1,219-image set. Top: per-agent Round-1 vs Round-3 crop and disease accuracy with the weighted consensus (red dashed line); peer-influence matrix (persuader rows, changed-agent columns). Bottom: per-agent combined accuracy R1 vs R3; agent-behavior breakdown (stubborn, R1→R2 change, flip-flop); and the impact of disease-verdict changes (helped vs hurt). The consensus is essentially unchanged by debate, while Qwen-VL improves markedly and is the most-persuaded agent.

The headline PANDA debate uses GPT-4.1, Qwen-VL, and Grok-4.20-Fast, selected from the VIDA stage based on diagnostic performance while maintaining model diversity, over all 1,219 images. We separately analyze (i) the effect of debate on the weighted ensemble verdict, (ii) the effect on each individual agent, and (iii) the directed peer influence underlying those changes. **Figure 3** provides a visual overview of all three.

#### 5.2.1 Consensus is anchor-bound

**Table 6** compares the pre-debate weighted baseline (computed from the same agents’ Round-1 predictions with identical weights) against the post-debate Round-3 consensus. McNemar’s test finds *no* significant change on any axis: Combined 0.359 → 0.360 (*p* = 1.000), crop 0.835 → 0.829 (*p* = 0.358), disease 0.436 → 0.436 (*p* = 0.915). The final consensus (Combined 0.360) is statistically indistinguishable from GPT-4.1’s standalone VIDA performance (0.366). Although debate induces substantial revisions in individual agent predictions, these changes largely cancel out at the ensemble level, resulting in a stable consensus that remains effectively unchanged from the pre-debate baseline.

**Table 6:** PANDA ensemble consensus before (weighted R1 baseline) and after (R3) debate, panel {GPT4.1, Qwen-VL, Grok-4.20-Fast}, *N* = 1,219.

Metric	Pre	Post	Δ	*b/c*	*χ*2	*p*
Crop	0.835	0.829	−0.007	33/25	0.845	0.358
Disease	0.436	0.436	+0.000	44/44	0.011	0.915
Combined	0.359	0.360	+0.001	47/48	0.000	1.000

McNemar’s test (continuity-corrected) finds no significant change on any axis.

#### 5.2.2 Individual agents change dramatically

The static consensus conceals substantial movement underneath (**Table 7**). The open-source agent Qwen-VL improves sharply through debate (Combined 0.183→0.304; Wilcoxon *p <* 0.0001), revising its disease verdict on 575 of 1,219 images with a strongly net-positive effect (159 corrected versus 37 degraded, net +122). GPT-4.1 edges up non-significantly (+0.010, *p* = 0.134) and Grok is essentially static (−0.003, *p* = 0.248) while remaining 98.7% stubborn. Thus debate’s benefit is concentrated in the weakest participant, exactly the agent a lighter open-source deployment would rely on but those individual gains are absorbed by the weighting and do not lift the ensemble (**Table 6**).

**Table 7:** Per-agent Combined accuracy from R1 to R3, with Wilcoxon signed-rank test, stubbornness (% of images where the agent never revised its disease verdict), and flip-flop rate (% changed then reverted).

Agent	R1	R3	Δ	Wilcoxon *p*	Stub.%	FF%
GPT-4.1	0.366	0.376	+0.010	0.134	85.8	0.5
Qwen-VL	0.183	0.304	+0.121	*<* 0.0001	42.2	12.1
Grok-4.20-Fast	0.326	0.322	−0.003	0.248	98.7	0.0

#### 5.2.3 Persuasion is asymmetric and decoupled from accuracy

**Figure 4:**
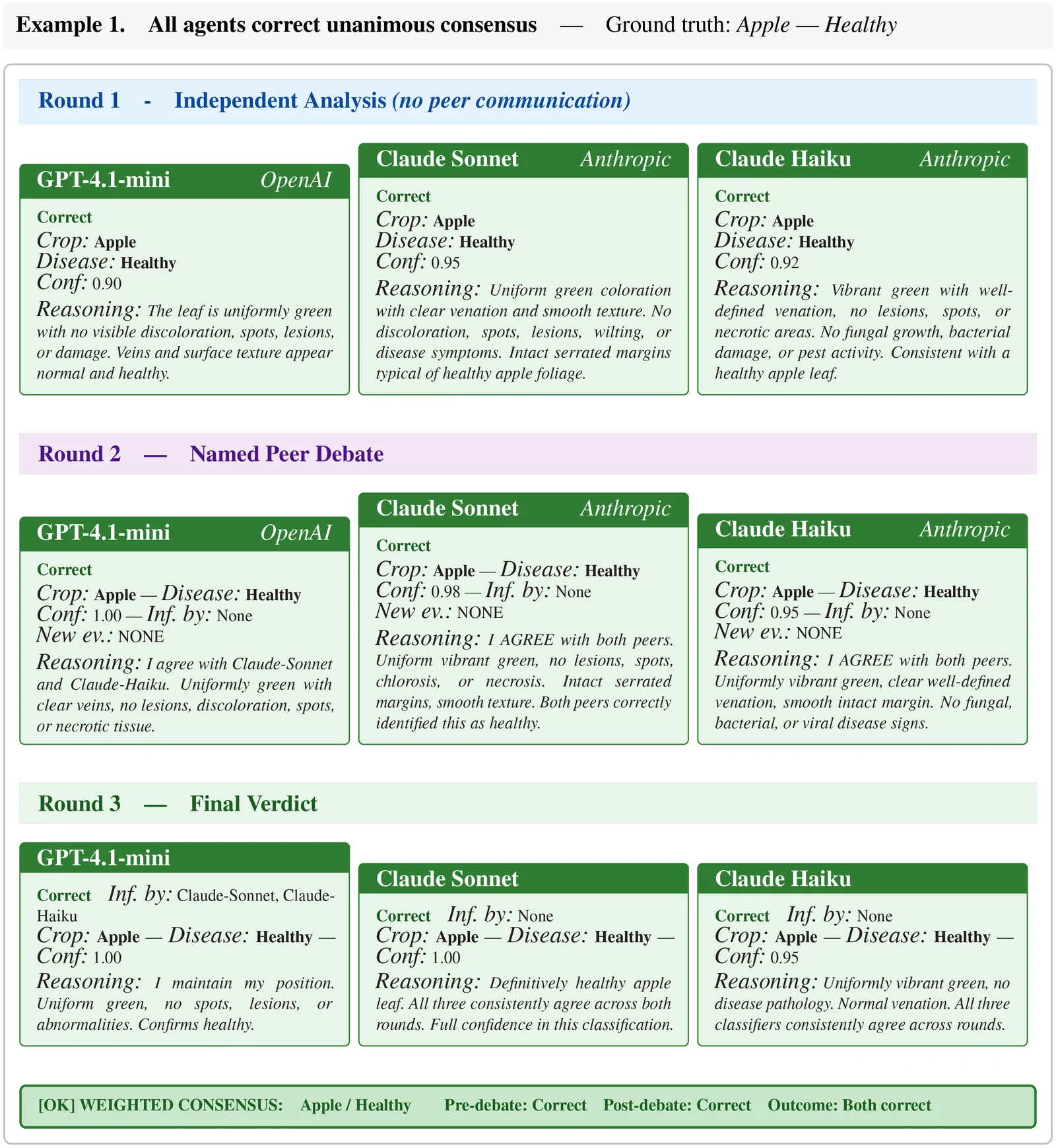
Representative PANDA debate transcript showing unanimous correct agreement. All three agents correctly classify the image as Apple/Healthy across independent analysis, named debate, and final verdict rounds.

**Figure 5:**
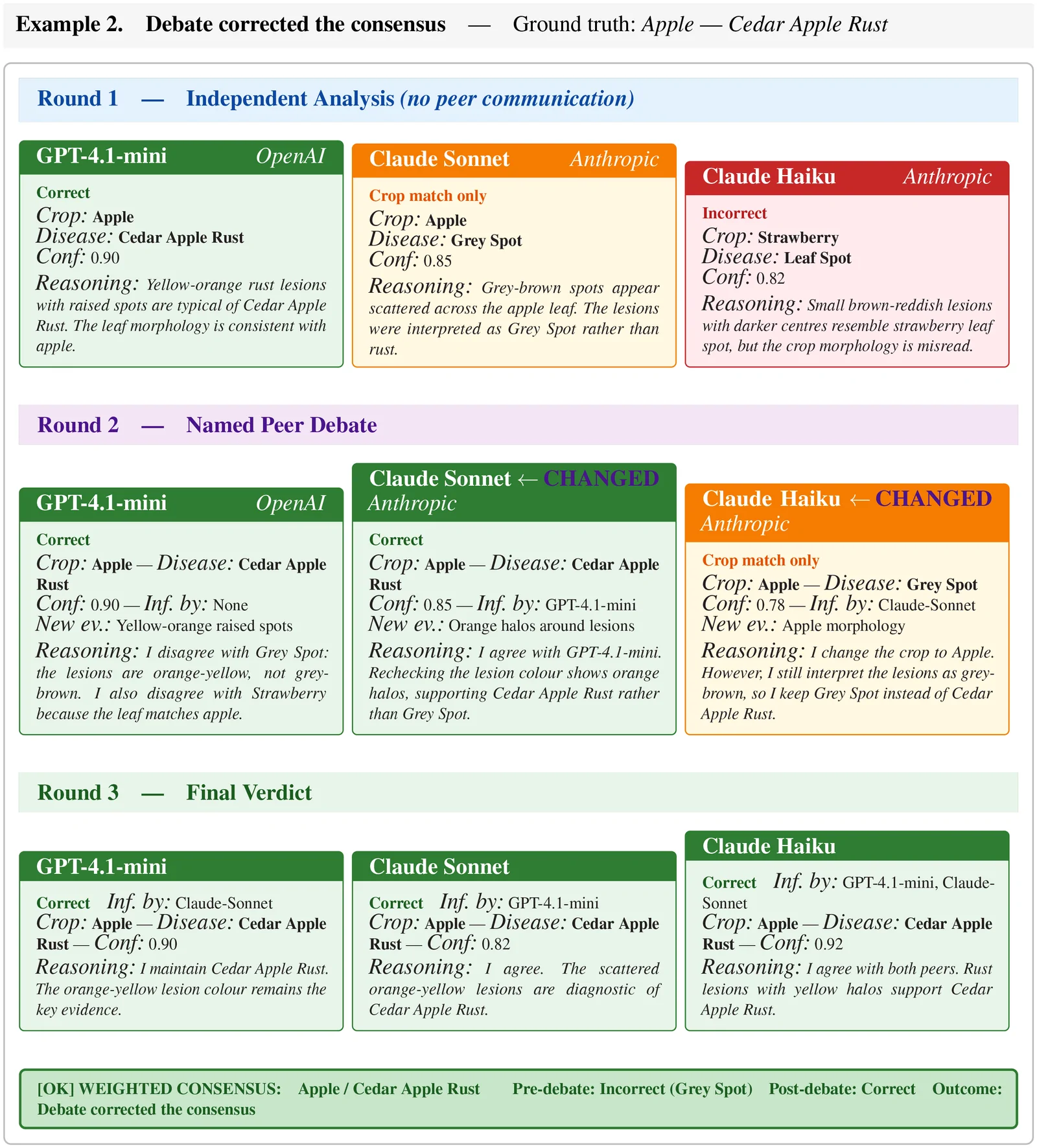
Representative PANDA debate transcript showing a debate-corrected consensus. Initial predictions disagree, but named peer debate helps the agents converge on the correct Apple/Cedar Apple Rust diagnosis.

**Figure 6:**
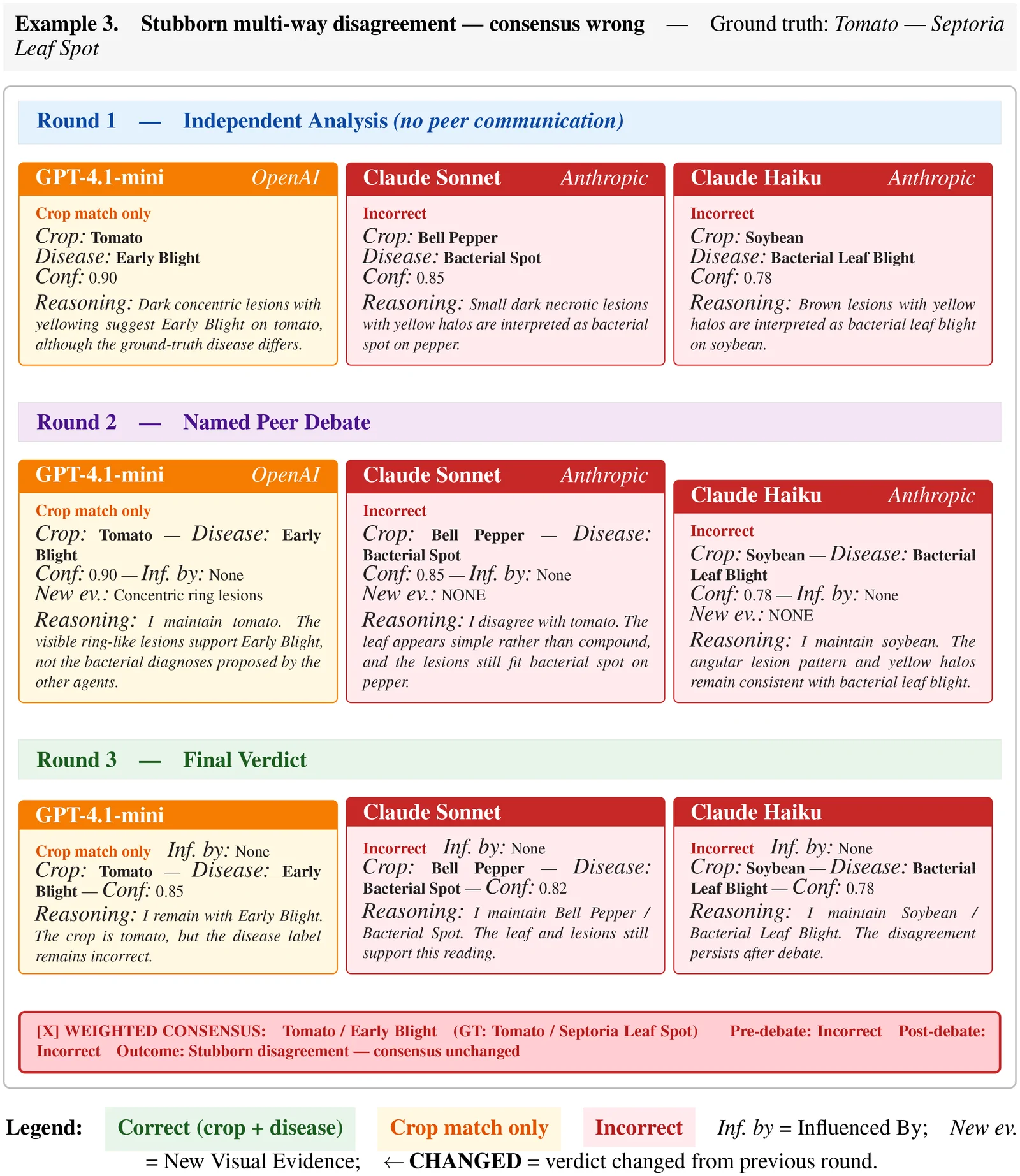
Representative PANDA debate transcript showing stubborn multi-way disagreement. The agents maintain conflicting diagnoses across debate rounds, and the weighted consensus remains incorrect despite peer exchange.

**Table 8** counts how often each agent’s reasoning was cited as the reason a peer changed its verdict. The matrix is strikingly asymmetric: GPT-4.1 persuades Qwen-VL 882 times, and Grok persuades Qwen-VL 324 times, while Qwen persuades others only rarely. Most revealing, Grok-4.20 is simultaneously highly persuasive toward Qwen and the most *stubborn* agent (revising in only 1.3% of cases) and among the least accurate of the three. Persuasive influence is therefore not a proxy for correctness a confident voice reshapes peers regardless of whether it is right. Representative debate transcripts illustrating the three qualitative outcomes of deliberation stable agreement, debate-corrected consensus, and stubborn disagreement are shown in **Figures 4**–**6**.

**Table 8:** Directed peer influence: number of times the row agent’s reasoning was cited as the source of the column agent’s verdict change (R2+R3 combined).

Persuader ↓/Target →	GPT-4.1	Qwen-VL	Grok-4.20-Fast
GPT-4.1	–	882	17
Qwen-VL	145	–	10
Grok-4.20-Fast	122	324	–

Persuasion is strongly asymmetric and decoupled from accuracy.

### 5.3 Agent-configuration ablation

To examine how the choice of debating agents affects outcomes, we conduct a panel-composition ablation study using four three-agent panels (PANDA-1 through PANDA-4) assembled from a pool of seven candidate agents. The ablation is evaluated on the original 490-image exploratory subset used during framework development, whereas the primary results reported elsewhere in this paper are based on the expanded 1,219-image evaluation set. By systematically varying which agents debate while holding the protocol fixed, this ablation isolates the effect of agent selection on debate dynamics and motivates our final panel choice. Its findings reinforce the anchor-bound behavior reported in Section 5.2.

#### 5.3.1 Accuracy before and after debate

**Table 9** reports pre- and post-debate Combined accuracy for the four configurations. PANDA-1 yields the largest improvement (Δ = +0.017, pre 0.367, post 0.384), with debate correcting the consensus on 18 images and degrading it on only 10 (**Table 10**). PANDA-2 shows a marginal improvement (Δ = +0.002) with near-equal helped/hurt counts (19 vs 18). PANDA-3 and PANDA-4 show no net change (Δ = 0.000); PANDA-3 notably operates at a lower baseline (0.286) because it excludes GPT-4.1. The contrast is the central result of this ablation: the two panels that move the consensus or sit at a high baseline both contain GPT-4.1, whereas the one panel that omits it neither improves nor reaches a competitive baseline. A dominant high-accuracy anchor is therefore necessary for debate to improve, or even sustain, the ensemble verdict.

**Table 9:** PANDA panel configurations evaluated on the original 490-image exploratory subset.

Config	Agents	Pre	Post	Δ
PANDA-1	GPT-4.1, GPT-4.1-mini, Grok-4.20-Fast	0.367	0.384	+0.017
PANDA-2	GPT-4.1, GPT-4.1-mini, Claude Sonnet	0.361	0.363	+0.002
PANDA-3	GPT-4.1-mini, Claude Sonnet, Claude Haiku	0.286	0.286	+0.000
PANDA-4	GPT-4.1, Grok-4.20-Fast, Claude Sonnet	0.369	0.369	+0.000

Pre/post Combined Accuracy is reported for each configuration; Δ denotes post − pre.

**Table 10:** McNemar test for pre- vs post-debate combined accuracy (N = 490, exploratory subset).

Config	Pre	Post	Δ	*b*	*c*	*χ*2	Sig.
PANDA-1	0.367	0.384	+0.017	10	18	1.750	n.s.
PANDA-2	0.361	0.363	+0.002	18	19	0.000	n.s.
PANDA-3	0.286	0.286	+0.000	—	—	—	n.s.
PANDA-4	0.369	0.369	+0.000	—	—	—	n.s.
PANDA-2 crop	0.820	0.790	-0.031	20	5	7.840	∗∗

*b* = images where debate degraded a correct consensus; *c* = images where debate corrected an incorrect one. Exact *b*/*c* for PANDA-1/2 from raw results; PANDA-3/4 estimated. No configuration is significant for combined accuracy; PANDA-2 shows a significant *crop* decline.

^∗∗^ *p <* 0.01; n.s. = not significant. PANDA-3/4 *b*/*c* estimated.

McNemar tests (**Table 10**) show that no configuration reaches significance for Combined accuracy (*p >* 0.05); the modest gains are consistent with prior multi-agent debate work (Du et al., 2023), where improvements are typically incremental on hard tasks. A notable exception is the PANDA-2 *crop* accuracy decline (Δ = −0.031, *χ*^2^ = 7.84, *p* = 0.005), the only statistically significant accuracy change observed: a weaker agent (Claude-Sonnet) persuaded the stronger anchor (GPT-4.1) to revise a correct crop verdict, degrading performance on the very dimension where the anchor was strongest. This asymmetric degradation is an early, explicit instance of the persuasive-inconsistency risk quantified on the main run.

#### 5.3.2 Per-agent trajectories and anchor convergence

Although the ensemble consensus barely moves, individual agents benefit substantially from debate (**Table 11**): GPT-4.1-mini improves by +0.057 in PANDA-1 (Wilcoxon *p <* 0.001) and Claude-Sonnet by +0.151 in PANDA-2 (*p <* 0.001), while the strong anchor (GPT-4.1) and the stubborn Grok stay flat. A consistent pattern emerges around GPT-4.1-mini: in both panels it converges upward toward the anchor’s accuracy (0.294 → 0.351 in PANDA-1 and 0.294 → 0.345 in PANDA-2), yet it exerts no persuasive pull of its own. Its gains track the GPT-4.1 anchor rather than redirecting it, and because they merely echo a verdict the anchor already holds, they cancel at the ensemble level. More generally, weaker agents adopt the reasoning framing of stronger peers through named debate the same anchor-convergence effect observed for Qwen-VL on the main run (**Table 7**).

**Table 11:** Per-agent combined-accuracy trajectory across rounds (R1→R2→R3) for PANDA-1 and PANDA-2 (N = 490, exploratory subset).

Config	Agent	R1	R2	R3	Δ R1→R3	Wilcoxon *p*	Stubborn (R2)	Flip-flop
PANDA-1	GPT-4.1 Grok-4.20-Fast	0.369 0.302	0.390 0.298	0.388 0.302	+0.018 +0.000	0.210(n.s.) 1.000(n.s.)	89.4% 99.4%	0.6% 0.0%
	GPT-4.1-mini	0.294	0.341	0.351	+0.057	*<* 0.001^∗∗∗^	77.3%	2.4%
PANDA-2	GPT-4.1 GPT-4.1-mini	0.369 0.294	0.376 0.337	0.378 0.345	+0.008 +0.051	0.560(n.s.) 0.004^∗∗^	86.5% 75.1%	0.8% 2.9%
	Claude-Sonnet	0.169	0.300	0.320	+0.151	*<* 0.001^∗∗∗^	62.2%	2.9%

^∗∗^ *p <* 0.01; ^∗∗∗^*p <* 0.001; n.s.=*p >* 0.05. Wilcoxon signed-rank test on per-image R1 vs R3 correctness. Weaker agents improve significantly even when the ensemble consensus does not change.

#### 5.3.3 Peer influence across panels

**Figure 7:**
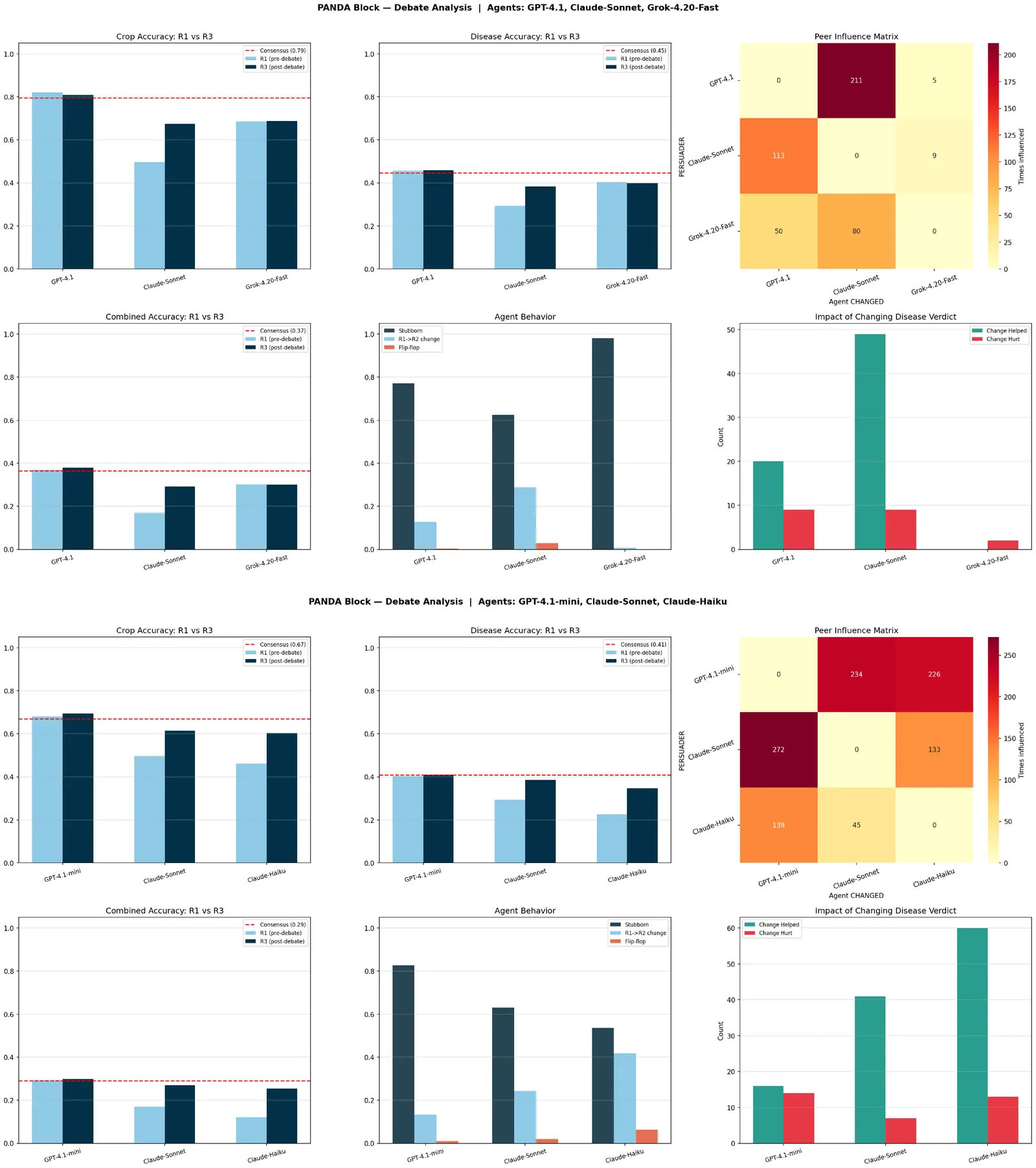
Debate analysis for PANDA-4 (top: GPT-4.1, Grok-4.20-Fast, Claude Sonnet) and PANDA-3 (bottom: GPT-4.1-mini, Claude-Sonnet, Claude-Haiku), *N* = 490. Each panel shows per-agent Round1 vs Round-3 accuracy (consensus as red dashed line), the peer-influence matrix, the agent-behavior breakdown (stubborn/changed/flip-flop), and the impact of disease-verdict changes (helped vs hurt). With a high-accuracy anchor (PANDA-4) the consensus holds at 0.369; without one (PANDA-3) the consensus stagnates at 0.286 despite strong individual gains.

**Figure 7** contrasts PANDA-4 (with GPT-4.1) and PANDA-3 (without it). In PANDA-4, GPT-4.1 dominates the influence matrix (211 persuasions) and the consensus holds at 0.369; in PANDA-3, Claude-Sonnet becomes the dominant persuader (272 persuasions) yet the consensus stagnates at 0.286 despite strong individual gains. The agents being persuaded are no more accurate than those persuading them, so individual improvements cancel at the ensemble level. This is the clearest statement of the ablation’s message: named debate improves individual reasoning under any configuration, but the ensemble improves only when a high-accuracy anchor is present to absorb that improvement rather than dilute it.

#### 5.3.4 Illustrative debate transcripts

**Figures 4**-**6** present representative debate transcripts for the three qualitatively distinct outcomes characterized in Section 5.2: a stable case in which all agents independently reach and maintain the correct diagnosis (**Figure 4**); a case in which named peer debate corrects an initially wrong consensus by propagating new visual evidence (**Figure 5**); and a failure case in which agents remain locked into conflicting diagnoses, yielding an incorrect post-debate consensus (**Figure 6**).

### 5.4 Semantic evaluation of reasoning convergence

**Figure 8:**
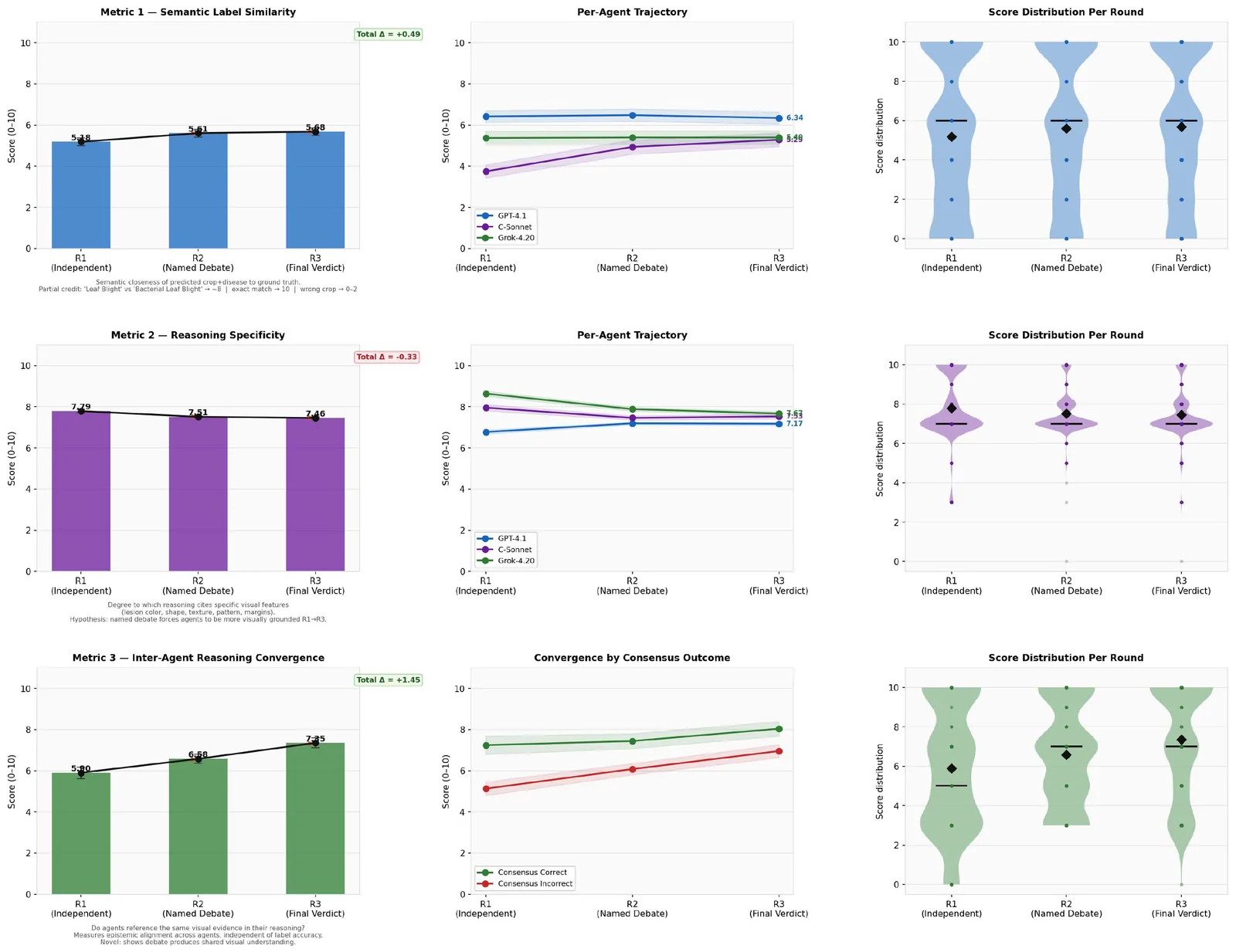
Semantic evaluation across debate rounds (PANDA-4 ablation, *N* = 490). Row 1: Semantic Label Similarity (SLS), semantic closeness of predicted labels to ground truth (Δ_R1→R3_ = +0.49). Row 2: Reasoning Specificity (RS), degree to which reasoning cites specific visual features (Δ_R1→R3_ = −0.33). Row 3: Inter-Agent Reasoning Convergence (IRC), degree to which agents reference the same visual evidence, independent of label accuracy (Δ_R1→R3_ = +1.45). For each metric, the left panel shows the round mean with 95% confidence intervals; the center panel shows per-agent trajectories (rows 1–2) or a split by consensus outcome (row 3); the right panel shows the score distribution as violin plots.

A central contribution of this framework is that it evaluates *how* agents reason, not only whether the final label is correct. We compute this semantic analysis on the PANDA-4 configuration from the agent-selection ablation (Section 5.3; *N* = 490), which provides a single, internally consistent evaluation setting across all three metrics. **Figure 8** summarizes Semantic Label Similarity (SLS), Reasoning Specificity (RS), and Inter-Agent Reasoning Convergence (IRC) across the debate rounds.

Three findings emerge. First, Semantic Label Similarity improves moderately across rounds (SLS: 5.18 → 5.68, Δ = +0.49); per-agent trajectories show GPT-4.1 holding steady near 6.34 while ClaudeSonnet and Grok-4.20-Fast converge upward toward it by Round 3 the anchor-convergence effect at the semantic level. Second, Reasoning Specificity declines slightly (RS: 7.79 → 7.46, Δ = −0.33), explained by a compositional shift: in later rounds part of each response is consumed by peer-addressing language (“I agree/disagree with [Agent]…”), reducing the budget for raw visual description a genuine tradeoff between social coherence and individual grounding. Third, and most novel, Inter-Agent Reasoning Convergence rises sharply (IRC: 5.80 → 7.25, Δ = +1.45), and images whose consensus was ultimately correct converge to a higher IRC (≈ 8.0) than those whose consensus was incorrect (≈ 7.0). This “IRCpredicts-correctness” relationship is the key semantic finding: genuine epistemic convergence agents citing the same visual evidence rather than merely adopting a peer’s label is a signal of a reliable diagnosis.

By construction, RS and IRC score whether agents cite specific visual evidence and whether they agree with one another, rather than whether they match any single reference verdict; reasoning convergence is therefore the primary signal this analysis reports. Validating these semantic scores against human agronomist judgments at larger scale is a natural extension.

## 6 Discussion

### 6.1 Debate helps agents, not the ensemble

The clearest result of this study is a dissociation between individual and ensemble effects. Performance-weighted consensus is bounded by the strongest anchor and does not improve through debate (**Table 6**), yet the weakest individual participant improves substantially (**Table 7**). For a practitioner, the implication is that multi-agent debate is most valuable when the goal is to raise the floor of a weak or inexpensive model, not to push the ceiling of an already-strong ensemble. This reframes “does debate help?” as “help whom?”.

### 6.2 Persuasive inconsistency as the central risk

The peer-influence analysis exposes a failure mode we term *persuasive inconsistency*: the agents that most reshape their peers are not the most accurate, and one of the most persuasive agents is also the most stubborn (**Table 8**). In a diagnostic setting this is dangerous a confidently wrong agent can pull a correct peer off its verdict purely through assertive reasoning. Our anti-sycophancy constraint (a verdict may change only when new visual evidence is explicitly cited) and the machine-parseable Influenced By field are designed to make this dynamic measurable and to dampen it, but the residual asymmetry shows the problem is not fully solved. Calibrating influence to demonstrated reliability is, in our view, the most important open problem for diagnostic multi-agent systems.

### 6.3 Open-source viability and API budget

Reproducibility and cost are practical barriers to deployment. Including two open-weight models and placing Qwen-VL in the main debate panel shows the framework does not depend on closed APIs, and Qwen-VL was the single largest beneficiary of debate an encouraging result for lighter setups. Cost nonetheless remains a real constraint: each image triggers up to three debate rounds across multiple commercial endpoints, and large hosted open models proved unreliable at production scale (dedicated endpoints required, empty responses in default thinking modes, timeouts), so they are best reserved for smaller or specialized roles. Our frozen-Round-1 design mitigates this by computing the expensive independent-diagnosis stage once and caching it, so debate configurations can be explored without repeating VIDA inference. Promising directions for further cost reduction include selective debate (triggering deliberation only on low-agreement images), distilling the debate behavior into a single model, and capping rounds adaptively when convergence is reached.

### 6.4 Fine-grained failure and the role of few-shot adaptation

The per-disease analysis (**Table 4**) localizes the remaining errors precisely: agents recognize the crop but not the specific lesion, and the lone zero-accuracy class (Target Spot) is a genuine fine-grained discrimination failure rather than a sampling artifact. Because scaling the sample already eliminated five of six earlier zeros, we do not view targeted few-shot exemplars as necessary to validate the zero-shot framework; rather, lightweight few-shot prompting for the handful of confusable classes is a natural refinement and a clear avenue for future work.

### 6.5 Limitations

Our evaluation uses curated single-leaf imagery; performance under field conditions cluttered backgrounds, occlusion, mixed infection, variable lighting remains untested. The framework is image source agnostic, so extending it to in-field photographs and to validation at our greenhouse facilities is a direct next step rather than a redesign. The semantic reasoning scores would further benefit from validation against human-expert judgments, which we leave to future work. Finally, although the judge is a non-participant in the main evaluation, it shares a provider with two debating agents; a fully cross-provider judge would further harden the evaluation, and the judge is implemented as a configuration variable to enable this.

## 7 Conclusion

We presented VIDA+PANDA, a zero-shot, heterogeneous multi-agent vision-language framework for crop disease diagnosis, and evaluated it on 1,219 images across 53 classes with a non-participant GPT-5 judge and the dataset identity withheld from prompts. Two findings stand out. First, structured peer debate does not improve the performance-weighted ensemble verdict, which remains bounded by its strongest anchor, yet it substantially improves the weakest individual agent an open-source model demonstrating that debate raises the floor rather than the ceiling. Second, and more cautionary, peer influence is strongly asymmetric and decoupled from accuracy: the most persuasive agent need not be the most correct or the most willing to update. This persuasive inconsistency is the principal risk of diagnostic multi-agent debate and must be controlled before such systems are trusted in practice. Alongside these dynamics, we introduced three semantic metrics (SLS, RS, IRC) and showed that inter-agent reasoning convergence tracks diagnostic correctness, offering a reasoning-level signal of reliability beyond label agreement. Future work targets field imagery, few-shot adaptation for the small set of fine-grained classes that remain unsolved, reliability-weighted influence to suppress persuasive inconsistency, and integration with a segmentation pipeline for greenhouse validation.

## Data Availability

The original contributions presented in the study are included in the article/supplementary material. Further inquiries can be directed to the corresponding author.
